# *IQSEC2*-related developmental and epileptic encephalopathy with a Rett-like phenotype: two cases with novel variants and a review of the literature

**DOI:** 10.3389/fped.2026.1786453

**Published:** 2026-05-14

**Authors:** Li Cheng, Bo Lei, Li Geng, Dan Li, Chaobin Zhou, Chengfang Yan, Qingtao Chen, Maoqiang Tian

**Affiliations:** 1 Department of Pediatrics, Anshun People's Hospital, Anshun, Guizhou, China; 2 Department of Pediatrics, Guanling County People's Hospital, Anshun, Guizhou, China; 3 Department of Pediatrics, Affiliated Hospital of Zunyi Medical University, Zunyi, Guizhou, China

**Keywords:** developmental encephalopathy, epileptic encephalopathy, *IQSEC2*gene, Rett-like syndrome, Rett syndrome

## Abstract

Rett syndrome, related to methyl-CpG binding protein *2* (*MECP2*)*,* primarily affects females, though recent studies have identified additional causative genes, such as IQ motif and SEC7 domain-containing protein 2 (*IQSEC2*), which mainly affect males and are characterized by prominent developmental encephalopathy. Variations in these genes produce clinical manifestations similar to Rett syndrome but do not fully align with it, posing diagnostic challenges. Currently, they are clinically categorized as Rett-like syndromes. The genotype–phenotype relationships in *IQSEC2*-associated Rett-like syndromes remain poorly characterized. Herein, we describe two brothers with a novel *IQSEC2* truncating variant and Rett-like syndrome manifestations and, alongside a synthesis of 38 published cases, provide insights into the genotype–phenotype correlations of *IQSEC2*. Two brothers with a novel *IQSEC2* variant presented with Rett-like syndrome, exhibiting developmental delay, microcephaly, refractory epilepsy, stereotypic hand movements, and absent speech. Their heterozygous mother displayed mild intellectual disability and late-onset epilepsy. Among the 38 published cases and combined with these siblings, male predominance emerged as a key feature. Regression—unlike in *MECP2*-related Rett syndrome—occurred inconsistently. We hypothesize that this may represent a phenotype of developmental arrest rather than true regression. All nine missense variants mapped to functionally critical domains of the IQSEC2 protein, whereas 82% of the truncating mutations clustered outside these regions. The patients with large duplications or deletions demonstrated more severe phenotypic variability. Thus, these genotype–phenotype correlations support routine *IQSEC2* screening in patients with unexplained developmental and epileptic encephalopathy and Rett-like syndrome. Missense and truncated mutations associated with Rett-like syndrome demonstrate a clustered distribution within the IQSEC2 protein. The genotypes and variant locations help explain the phenotypic variation in patients with *IQSEC2* variants.

## 1 Introduction

The IQ motif and SEC7 domain-containing protein 2 (*IQSEC2*, OMIM *300522) gene, located on Xp11.22, encodes a guanine nucleotide exchange factor critical for synaptic plasticity (1). IQSEC2 is a protein with 1,488 amino acids, containing the IQ-like, Sec7, PH, and PDZ-bm domains [IQSEC2 in UniProtKB search (684) | UniProt]. A pathogenic variant in *IQSEC2* causes X-linked neurodevelopmental disorders (OMIM #309530), ranging from intellectual disability to severe developmental and epileptic encephalopathy (2–4). Emerging evidence indicates that patients with *IQSEC2* mutations may resemble those with Rett syndrome (RS) caused by methyl-CpG binding protein *2* (*MECP2*) mutations (5–7), particularly due to shared characteristics such as regression, stereotypic movements, and epilepsy. Regression is a distinguishing feature of RS, which emphasizes the gradual loss of previously acquired skills after a certain age, including hand dysfunction, motor regression, and regression in social and cognitive abilities (8). These symptoms, when caused by a non-*MECP2* disorder, are referred to as Rett-like syndrome (RLS). To date, more than 80 genes have been reported to be associated with the RLS phenotype, and the number is steadily increasing (6). Nevertheless, *IQSEC2*-associated RLS remains poorly defined. Herein, we characterize two brothers with a novel *IQSEC2* truncating variant and RLS manifestations and, alongside a synthesis of published cases, aim to provide further insights into the genotype–phenotypic correlations of *IQSEC2*.

## 2 Case description

### 2.1 Case 1

**Figure 1:**
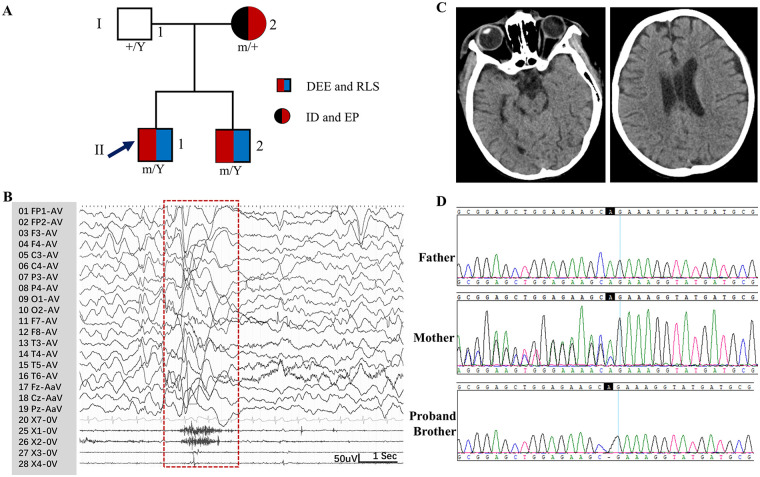
Clinical and genetic data of the patients with the *IQSEC2* variant. **(A)** Pedigrees of the cases with the *IQSEC2* variant and their corresponding phenotypes (DEE, developmental and epileptic encephalopathy; EP, epilepsy; ID, intellectual disability; RLS, Rett-like syndrome). **(B)** Video-electroencephalogram shows epileptic spasm (red dotted box) associated with a generalized suppression on the electroencephalogram of case 1. **(C)** Brain CT of case 2. **(D)** DNA sequence chromatograms of the *IQSEC2* variant.

A 9-year-old boy (Figure 1AII-1) presented with global developmental delay (head control at 5 months, independent sitting at 13 months) and seizure onset at 3 years old. Epilepsy evolved from epileptic spasms (Figure 1B) to generalized tonic-clonic seizures. His head circumference at birth was not documented but, at age 9, he exhibited microcephaly (head circumference of 42.1 cm, <−2 SD). Stereotypic hand-rubbing occurred at age 3 (see Supplementary Video). No significant facial dysmorphisms were observed. Diffuse hypotonia was noted. The patient also presented with significant feeding difficulties, including poor chewing and frequent choking, requiring supervision during meals. The interictal electroencephalogram (EEG) showed multifocal epileptiform activity and slow waves. Neuroimaging was unremarkable. Treatment with valproate (33 mg/kg/day) and carbamazepine (33 mg/kg/day) yielded poor seizure control.

### 2.2 Case 2

Case 1’s 7-year-old younger brother (Figure 1AII-2) showed more severe psychomotor developmental impairment, as he was non-ambulatory, non-verbal, and had monthly generalized seizures that began at age 2, partially controlled by levetiracetam (50 mg/kg/day) and oxcarbazepine (27 1 mg/kg/day). His head circumference at birth was not documented, but his head circumference at age 7 was 43.5 cm (< −2 SD), indicating microcephaly. Stereotypic hand movements were noted (see Supplementary Video). Brain computed tomography revealed cerebral volume loss and a cavum septi pellucidi (Figure 1C). The interictal EEG for this patient also revealed multifocal epileptiform activity.

**Figure 2:**
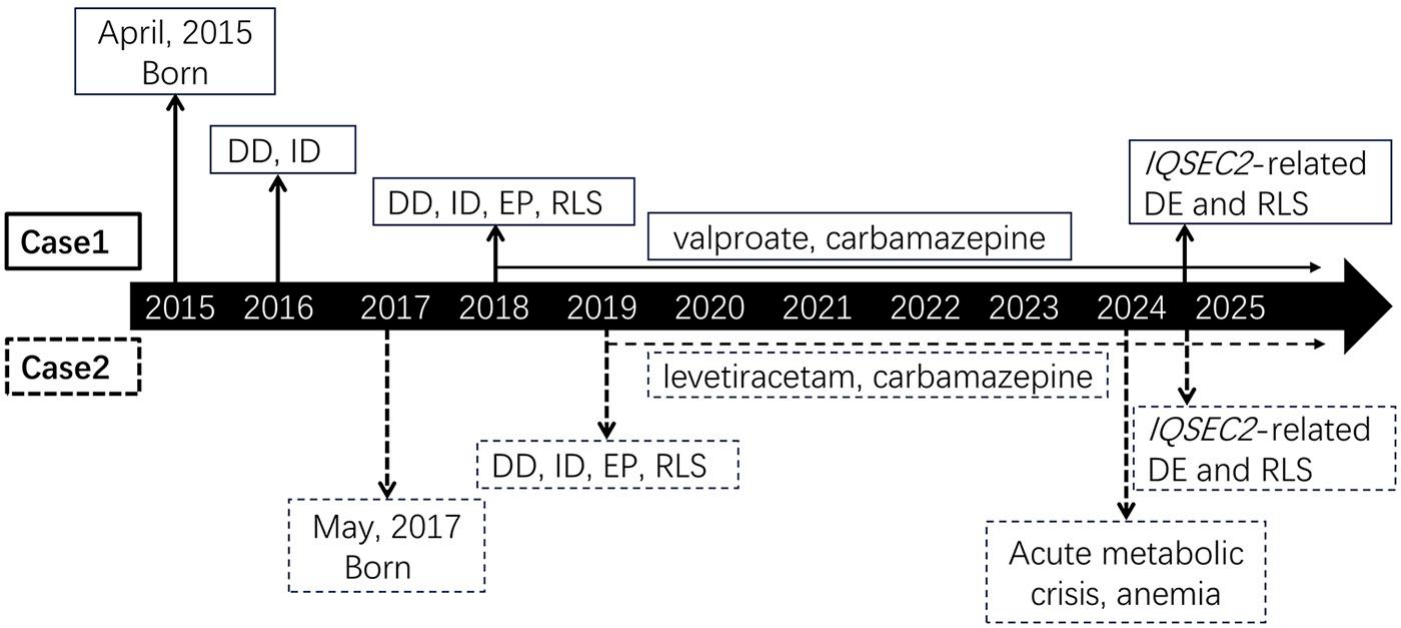
The timeline of diagnosis and treatment. DD, developmental delay; DE, developmental encephalopathy; EP, epilepsy; ID, intellectual disability; RLS, Rett-like syndrome.

Case 2 was admitted at age 7 following 1 week of vomiting, poor feeding, and lethargy. Upon hospitalization, the patient presented with severe dehydration and metabolic disturbances, including acidosis (HCO₃^−^ of 10.1 mmol/L, lactate of 9.7 mmol/L), anemia [hemoglobin (HGB) of 87 g/L], and hypokalemia (2.96 mmol/L). The patient's metabolic derangements resolved with symptomatic treatment, and the anemia improved. These laboratory test abnormalities were therefore considered secondary to the patient’s prolonged fasting and vomiting, which led to volume depletion and subsequently induced acidosis and electrolyte disturbances.

The 32-year-old mother of the siblings had mild intellectual disability (intelligence quotient was 68), delayed language development, and seizures since adolescence. No affected relatives were identified across three generations (*n* = 51). An inborn error of metabolism was suspected. Following consent from the family, trio whole-exome sequencing (WES) was performed. WES revealed a hemizygous *IQSEC2* frameshift variant (NM_001111125.2: c.3293delA) in both brothers that was heterozygous in the mother (Figure 1D). This variant leads to the premature termination of the IQSEC2 protein (p. Q1098Rfs*4). It is absent in gnomAD and meets the pathogenic criteria according to the American College of Medical Genetics and Genomics guidelines (PVS1 (Pathogenic very strong evidence 1 (null variant in a gene where loss-of-function is a known mechanism of disease)), PM2 (pathogenic moderate evidence 2 (absent from controls)), and PP1 (pathogenic supporting evidence 1 (cosegregation with disease in multiple affected family members))) (9). This family has been diagnosed with an *IQSEC2*-related disorder. The clinical course of the patient is summarized in Figure 2.

## 3 Discussions

**Figure 3:**
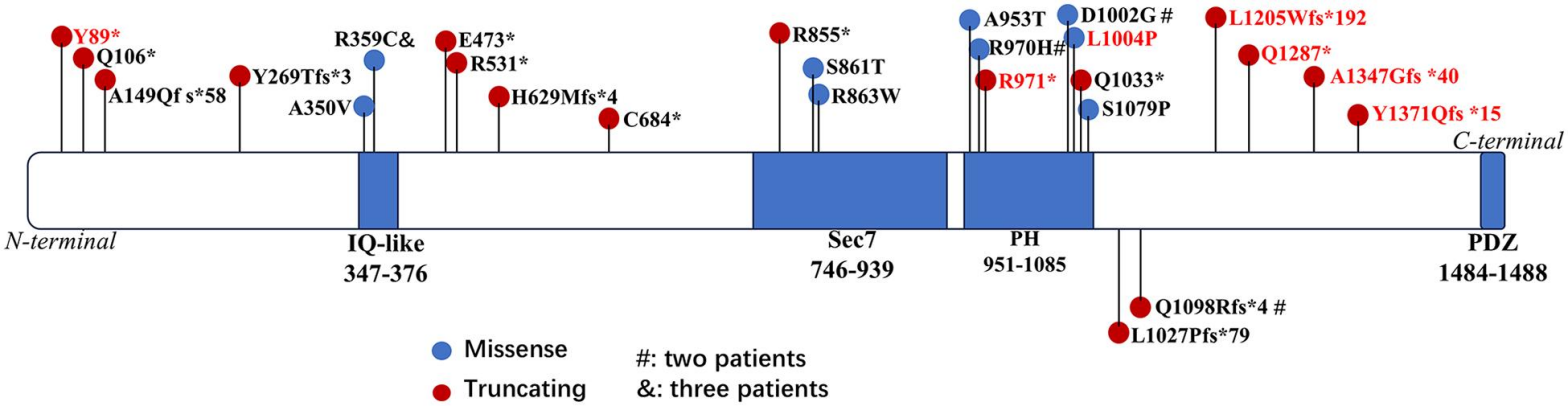
Schematic diagram of *IQSEC2* variants in patients with RLS. Red text: female; black text: male.

We reviewed the literature. After excluding patients for whom genetic information could not be obtained and those with unconfirmed symptoms of RLS, a total of 38 patients carrying *IQSEC2* variants and presenting with RLS have been reported (1, 5, 10–15). With the inclusion of the two new cases in this report, a total of 40 patients with *IQSEC2* variants and RLS have been identified, as outlined in the Supplementary Table. RLS-related variants span the entire IQSEC2 protein and the molecular subregional locations are summarized in Figure 3.

The triad of intellectual disability (100%), language impairment (97.5%), and stereotypies (82.5%) emerged as universal features, thus mirroring core RS features but with notable differences. Our findings highlight a male predominance (85%, 34/40) in *IQSEC2*-associated cases with RLS, while classic RS due to *MECP2* mutations predominantly affects female patients (6, 16). The large comparative study by Mignot et al. further corroborates this, finding that while the number of affected female patients is comparable, male patients generally exhibit more severe developmental delay and intellectual disability, with epilepsy being more common, more refractory, and having an earlier onset (3). Unlike *MECP2*-related RS, regression is not explicitly documented here, suggesting that *IQSEC2* variants may drive a developmental arrest phenotype rather than postnatal regression. Thus, the inclusion of two adult patients (ages 34 and 44 years) (5) underscores the lifelong burden of this disorder, with recurrent seizures and persistent intellectual and communicative deficits.

Our previous study revealed that the molecular subregional location of the variant was a crucial factor in determining its pathogenicity and was linked to phenotypic severity (17). RLS-related variants span the entire IQSEC2 protein (Figure 3). We analyzed all 26 single nucleotide variations (33 patients) and found that all nine missense variants localize to functionally critical domains (i.e., the IQ-like, Sec7, and PH domains), while 82% (14/17) of the truncating mutations (nonsense and frameshift) clustered in non-domain regions, a pattern consistent with observations by Radley et al. (4) and echoing the finding by Mignot et al. that pathogenic variants predominantly lead to protein truncation and are scattered throughout the brain-specific long transcript (3). This clustering suggests a significant subregional effect of *IQSEC2*. While these findings are compelling, they are based on a limited number of cases and require further validation. We hypothesize that these patterns may indicate a dose-dependent effect of *IQSEC2* dysfunction. The high probability of being loss-of-function intolerant (PLI=1) for IQSEC2 further supports this hypothesis, as a higher PLI indicates a greater dosage dependence of the gene. Missense variants in the functional regions may lead to alterations in the *IQSEC2* protein that are comparable to those caused by truncating variants in non-functional regions.

Large duplications/deletions (Cases 34–40) exhibited broader phenotypic severity, including microcephaly and behavioral abnormalities, potentially due to disruption of adjacent genes (e.g., *BRWD3*, *TSPYL2*, and *KDM5C*) (15, 17). Furthermore, our study suggests a predominance of *de novo* variants (29/40, 72.5%). The study by Mignot et al. refines this finding, noting that *de novo* variants predominate in female patients, whereas in male patients, approximately one-third are inherited, and most missense variants are inherited (3). The variable expressivity among female carriers, along with the discordant phenotype in monozygotic twins and possible gonadal mosaicism, was reported by Radley et al. (4). The variable expressivity among female carriers underscores the importance of thorough family counseling. This study has several limitations. The sample size of cases in this study was small. The direct functional effects of the variants were not examined. Further research with more cases is needed to clarify the genotype–phenotype correlation of *IQSEC2*.

## 4 Conclusions

In summary, this study suggests that *IQSEC2*-related disorders may be a new disease entity rather than a continuum of the RS spectrum. These findings advocate for routine *IQSEC2* screening in patients with “atypical RS” features. Missense and truncated mutations associated with RLS demonstrated a clustered distribution within the IQSEC2 protein. This observed clustering, along with the genotype, may help explain the phenotypic variation of patients with *IQSEC2* variants. However, these conclusions should be considered preliminary, and further studies with larger cohorts and functional experiments are necessary to confirm these associations.

## Data Availability

The datasets presented in this study are not publicly available due to privacy/ethical restrictions but are available fromthe corresponding author upon reasonable request.

## Supplementary material

SUPPLEMENTARY VIDEO

Stereotypic hand movements in case 1 and case 2.

SUPPLEMENTARY TABLE

Summary of 38 published cases plus the two new cases with IQSEC2 variants and Rett-like syndrome.
