# Supplementary material for: IQSEC2-related developmental and epileptic encephalopathy with a Rett-like phenotype: two cases with novel variants and a review of the literature
Source: Front Pediatr. 2026 May 14;14:1786453. doi: 10.3389/fped.2026.1786453 (PMC13216230; doi:10.3389/fped.2026.1786453)
Supplement: Supplementary file 2 [file Table1.docx]

**Supplementary Table** Data of patients with *IQSEC2*-related neurodevelopmental disorders with Rett like syndrome

| Cases | Reference | age | Sex | Inheritance | Variants | Domains | Presentations |
| --- | --- | --- | --- | --- | --- | --- | --- |
| 1 | Lopergolo D ^3^ | 7 years | F | De novo | c. 267C>G; p. Y89* | Non-Domain | ID\stereotypies\developmental and language delay |
| 2 | Liu X ^6^ | 5 years | M | De novo | c.316C>T; p.Q106* | Non-Domain | Developmental delay\language delay\epilepsy |
| 3 |  | 4 years | M | Maternal | c.443_444dup; p. A149Qfs*58 | Non-Domain | Developmental delay\language delay\epilepsy\ID |
| 4 |  | 2 .5 years | M | De novo | c.804del; p. Y269Tfs*3 | Non-Domain | Developmental delay\epilepsy\stereotypies |
| 5 | Lopergolo D ^3^ | 20 months | M | Maternal | c.999+8A>G | / | ID\ developmental delay\language delay |
| 6 | Zerem A^1^ | 2.5 years | M | De novo | c.1049C>T; p. A350V | IQ-like | Absence of language\ID\epilepsy |
| 7 | Liu X ^6^ | 2.5years | M | De novo | c.1075C>T; p. R359C | IQ-like | Developmental delay\language delay\epilepsy |
| 8 | Lopergolo D ^3^ | 34 years | M | De novo | c.1076G>A; p. R359H | IQ-like | Developmental delay\language delay\ID\ epilepsy |
| 9 |  | 44 years | M | De novo | c.1076G>A; p. R359H | IQ-like | Developmental delay\language delay\ID\epilepsy |
| 10 | Zerem A^1^ | 2.9 years | M | De novo | c.928G>T; p. E310X | Non-Domain | Developmental delay\language delay \epilepsy |
| 11 | Liu X ^6^ | 2years | M | Denovo | c.1417G>T; p. E473* | Non-Domain | Speech dysfunction\ID\epilepsy |
| 12 | Lopergolo D ^3^ | 15 years | M | De novo | c.1591C>T; p. R531* | Non-Domain | Language delay\ID\stereotypies |
| 13 |  | 7 years | M | De novo | c.1881delC; p. H629Mfs*4 | Non-Domain | Absent language\developmental delay\ID\stereotypies |
| 14 | Gandomi SK ^7^ | 3 years | M | De novo | c.2052_2053delCG; p. C684* | Non-Domain | Language delay\stereotypies\epilepsy |
| 15 | Tran F ^8^ | 3 years | M | De novo | c.2563C>T; p. R855* | Sec7 | Delay development\ID\stereotypies |
| 16 | Zerem A^1^ | 5.5 years | M | De novo | c.2582G>C; p. S861T | Sec7 | ID\epilepsy\stereotypies\absence of language |
| 17 |  | 14 years | M | Maternal | c.2587C>T; p. R863W | Sec7 | Language delay\Epilepsy\ID |
| 18 | Shoubridge C ^9^ | 15 years | M | Unknown | c.2857G > A; p. A953T | PH | Seizures\non-verbal\ID |
| 19 |  | 2 years | M | Maternal | c.2909G>A; p. R970H | PH | Absent language\stereotypies\developmental delay |
| 20 |  | 4 years | M | Familial | c.2909G > A; p. R970H | PH | Absent speech\ID\stereotypies |
| 21 |  | 8 years | F | De novo | c.2911C>T; p. R971* | PH | Developmental delay\absence of language\ID |
| 22 |  | 5 months | M | Maternal | c.3005A>G; p. D1002G | PH | Developmental delay\language delay\epilepsy |
| 23 |  | 6 months | M | Maternal | c.3005A>G; p. D1002G | PH | Seizures\language delay\ID\stereotypies |
| 24 | Lopergolo D ^3^ | 11 years | F | De novo | c.3011T>C; p. L1004P | PH | Developmental delay\absence of language\ID |
| 25 | Zerem A^1^ | 10 years | M | De novo | c.3097C >T; p. Q1033* | PH | ID\stereotypies\epilepsy\absence of language |
| 26 | Liu X ^6^ | 3.5 years | M | De novo | c.3235T>C; p. S1079P | PH | Developmental delay\language delay \epilepsy\ID |
| 27 | Baladron B ^10^ | 15 years | M | De novo | 3300dup; p. L1027Pfs*79 | Non-Domain | Language delay\ID\ stereotypies |
| 28 | Present study | 9 years | M | Maternal | c.3293delA; p. Q1098Rfs*4 | Non-Domain | ID\stereotypies\epilepsy\absence of language |
| 29 |  | 7 years | M | Maternal |  | Non-Domain | ID\stereotypies\epilepsy\absence of language |
| 30 | Lopergolo D ^3^ | 8 years | F | De novo | c.3613_3613 delC; p. L1205Wfs*192 | Non-Domain | ID \language delay\stereotypeis |
| 31 |  | 2 years | F | De novo | c.3859C>T; p. Q1287* | Non-Domain | ID\ language delay\epilepsy\stereotypeis |
| 32 |  | 8 years | F | De novo | c.4039dup; p. A1347Gfs *40 | Non-Domain | Language, developmental delay\epilepsy\stereotypies |
| 33 |  | 8 years | F | De novo | c.4110_4111del; p. Y1371Qfs *15 | Non-Domain | ID\epilepsy\stereotypies |
| 34 | Zerem A^1^ | 5 years | F | De novo | T (X; 20) (p11.2; q11.2) | / | Developmental, language delay \epilepsy |
| 35 |  | 6 years | M | De novo | hg19: g.53283513–53325284 duplication | / | ID\stereotypies\ epilepsy\ absent language\ microcephaly |
| 36 | Moey C ^11^ | 3 years | M | De novo | hg19: g.52954520-53315542 duplication | / | Language and developmental delay\behavioral and social problems |
| 37 |  | 14 years | M | Maternal | hg19: g.52911287-53315010 duplication | / | ID \developmental delay\epilepsy |
| 38 | Lopergolo D ^3^ | 7 years | M | De novo | GRCh37: g.53270956 53296256  duplication | / | ID\stereotypies\absence of language\developmental delay |
| 39 |  | 19 years | F | De novo | GRCh37: g.52954520-53394275  deletion | / | ID\stereotypies\language delay\developmental delay |
| 40 |  | 14 years | M | De novo | GRCh37: g53300438 53341868  duplication | / | ID\stereotypies\absent language\developmental delay |

Previously published single nucleotide mutations in *IQSEC2* were aligned to the reference sequence NM_001111125.

F: female; ID: intellectual disability; M: male.
